# Apolipoprotein E gene polymorphism and the risk of cardiovascular disease and type 2 diabetes

**DOI:** 10.1186/s12872-019-1194-0

**Published:** 2019-09-14

**Authors:** Sudong Liu, Jing Liu, Ruiqiang Weng, Xiaodong Gu, Zhixiong Zhong

**Affiliations:** 1 Clinical Core Laboratory, Meizhou People’s Hospital (Huangtang Hospital), Meizhou Hospital Affiliated to Sun Yat-sen University, No 63 Huangtang Road, Meijiang District, Meizhou, 514031 People’s Republic of China; 2 Guangdong Provincial Engineering and Technological Research Center for Molecular Diagnostics of Cardiovascular Diseases, No 63 Huangtang Road, Meijiang District, Meizhou, 514031 People’s Republic of China; 3 Provincial Key Laboratory of Precision Medicine and Clinical Translational Research of Hakka Population, No 63 Huangtang Road, Meijiang District, Meizhou, 514031 People’s Republic of China; 4 Center for Precision Medicine, Meizhou People’s Hospital (Huangtang Hospital), Meizhou Hospital Affiliated to Sun Yat-sen University, No 63 Huangtang Road, Meijiang District, Meizhou, 514031 People’s Republic of China; 5 Center for Cardiovascular Diseases, Meizhou People’s Hospital (Huangtang Hospital), Meizhou Hospital Affiliated to Sun Yat-sen University, Meizhou, 514031 People’s Republic of China

**Keywords:** Apolipoprotein E polymorphism, Type 2 diabetes mellitus (T2DM), Cardiovascular disease (CVD)

## Abstract

**Background:**

The role of apolipoprotein E gene (APOE) in lipid metabolism has been well established, and APOE is associated with the risk of cardiovascular disease (CVD) and diabetes mellitus (DM). However, the relationship between APOE polymorphisms and type 2 diabetes (T2DM) with or without CVD remains unclear.

**Methods:**

In this cross-sectional study, a total of 924 participants including 211 controls (CVD-T2DM-), 247 T2DM patients with CVD (CVD-T2DM+), 232 CVD patients without T2DM (CVD + T2DM-) and 234 T2DM patients with CVD (CVD + T2DM+), were genotyped using chip platform. The association between APOE polymorphisms and T2DM patients with or without CVD was analyzed by univariable and multivariable logistic analysis.

**Results:**

The present study showed that the frequency of E3/E4 increased in T2DM patients with CVD (*p* < 0.01). The ε4 allele was higher in CVD patients without T2DM (*p* < 0.01) and T2DM patients with CVD (*p* < 0.01) as compared with the controls.

**Conclusions:**

The subjects carrying ε4 allele have increased risk of CVD and T2DM, and exhibit higher level of lipid profiles.

## Background

Type 2 diabetes mellitus (T2DM) is a complex metabolic disease, in which insulin resistance and beta cell damage cause hyperglycemia [1]. The incidence of T2DM increased rapidly because of the increased average life expectancy, increased prevalence of obesity and westernization of lifestyles in developing countries. However, long-term complications of T2DM are main causes of morbidity and mortality [2, 3]. T2DM is also a major independent risk factor for cardiovascular disease (CVD). CVD represents a leading health problem around the world [4]. Studies showed that people with diabetes have two to four times of propensity to develop coronary artery disease (CAD) and myocardial infarction (MI) [4, 5]. In fact, about 70% of T2DM patients aged ≥65 died of CVD, and the new T2DM patients exhibited the equal risk to CVD as patients with previous myocardial infarction [6]. To date, some studies have demonstrated that the interaction between T2DM and cardiovascular risk supported the gradual progression of vascular injury, which subsequently leads to atherosclerosis [7]. Several other risk factors towards CVD have been identified, such as dyslipidemia, oxidative stress, smoking, alcohol consumption, and genetic factors [7, 8]. Dyslipidemia or lipoprotein abnormalities may aggravate microvascular and macrovascular complications and promote atherosclerosis in T2DM patients [9, 10].

Apolipoprotein E is essential in the formation of chylomicrons, very low density and high density lipoprotein (HDL) and is involved in lipid metabolism, transport and digestion [11]. APOE is polymorphic, consisting of three common alleles, namely epsilon 2 (ε2), epsilon 3 (ε3) and epsilon 4 (ε4), and six different genotypes (E2/E2, E2/E3, E2/E4, E3/E3, E3/E4 and E4/E4) [12]. APOE polymorphism seemed to have some effect on patients with cardiovascular disease [13]. Previous studies also showed that ε4 allele was associated with an increased risk of CAD in T2DM patients [8, 14]. In the present study, we investigated the association of APOE polymorphism with T2DM and CVD in Hakka population in south China, as well as its effect on plasma lipid levels.

## Methods

### Study population

The subjects were recruited from cardiovascular clinic and endocrinology clinic of Meizhou People’s Hospital. The study was approved by the Ethics Committee of Meizhou People’s Hospital (NO: MPH-HEC 2017-A-29). Written informed consent was obtained from each patient. Genetic family history, medical history, and lifestyle habits were collected from clinical records. Additional clinical data and biochemical data were obtained from clinical and laboratory tests such as blood lipids, systolic blood pressure (SBP) and diastolic blood pressure (DBP). Dyslipidemia is defined if either of the following condition is met: (i) serum LDL-C > 130 mg/dL; (ii) serum TG > 150 mg/dL; (iii) serum TC > 200 mg/dL; (iv) serum HDL-C < 40 mg/dL. Hypertension is defined as SBP/DBP above 140/90 mmHg. Diabetes is defined as fasting blood glucose ≥6.11 mmol/L and HbA1c ≥ 6.5%.

The control group consist of 211 subjects whose fasting blood glucose is < 6.11 mmol/L. The exclusion criteria included hyperlipidemia, hypertension, cardiovascular diseases, diabetes, liver and kidney diseases, metabolic disorders and autoimmune diseases.

T2DM patients without CVD consist of 247 whose fasting blood glucose is≥6.11 mmol/or taking oral diabetes medication without any history or signs of CVD. Exclusion criteria included malignant tumors, liver and kidney diseases, and autoimmune diseases.

CVD patients without T2DM included 232 patients whose fasting blood glucose is < 6.11 mmol/L. Exclusion criteria included kidney disease, liver disease, endocrine disease, metabolic disorders, and autoimmune diseases.

T2DM patients with CVD included 234 patients whose fasting blood glucose ≥6.11 mmol/L or taking oral diabetes medication and any form of cardiovascular disease. Exclusion criteria included nephropathy, liver and endocrine disease, metabolic disorders and autoimmune diseases.

### Measurement of laboratory parameters and APOE genotyping

Clinical data such as gender, age, diabetes, obesity, hypertension and family history was collected prospectively. The fasting lipid profiles and blood glucose were examined the next morning after admission by selective solubilization method (AU5400 analyzer, Beckman Coulter, CA, USA). Blood samples were stored in 2 ml vacuum tubes containing EDTA. According to the manufacturer’s protocol, Genomic DNA was extracted from the samples using QIAamp DNA Blood Mini Kit (Qiagen Germany) and quantified by NanoDrop 2000 TM spectrophotometer (Thermo Fisher Scientific, Waltham, MA, USA). The single nucleotide polymorphisms of APOE gene were detected by a commercially available kit (Zhuhai Zhongzhi Biotechnology Co., Ltd.), According to the following scheme, Polymerase chain reaction (PCR) was performed: 50 °C for 2 min, pre-denaturation at 95 °C for 15 min, followed by 45 cycles of denaturing at 94 °C for 30s, annealing at 65 °C for 45 s. Detection of amplified products (Sinochips Bioscience Co., Ltd., Zhuhai, Guangdong, China) by using APOE genotyping kit (gene chip analysis).

### Statistical analysis

The data were analyzed with IBM SPSS 20.0 software (Social Science Statistics Software Package). Data were assessed for normality using Kolmogorov–Smirnov test. Continuous data were expressed as mean ± standard deviation (SD) or median ± interquartile range based on the normality of distribution. Groups were compared using Student’s *t*-test or Mann-Whitney test. Categorical variables were expressed as frequency and compared using Chi-square (χ^2^) test or Fisher’s exact test. The association between disease and risk factors was analyzed by univariate logistic regression analysis with the determination of adjusted odds ratio (OR). All tests were two-sided and a *p* value < 0.05 was considered significant.

## Results

### The characteristics and biochemical variables of the study population

The demographic and clinical biochemical data are summarized in Table 1. The study included 924 subjects. These subjects were divided into four groups which were CVD-T2DM- (*n* = 211), CVD-T2DM+ (*n* = 247), CVD + T2DM- (*n* = 232) and CVD + T2DM- (*n* = 234). Laboratory data of patients with CVD or T2DM, such as blood glucose, blood lipids, SBP and DBP, were significantly higher than those of the control group. The incidence of hypertension (*p* < 0.01) and dyslipidemia (*p* < 0.01) was lower in CVD patients without T2DM compared with T2DM patients. The incidence of hypertension (*p* < 0.01) and dyslipidemia (*p* < 0.01) was lower in CVD patients with T2DM compared CVD patients without T2DM. The frequency of smoking was significantly higher in CVD patients than in T2DM patients (*p* < 0.01). Significant higher levels of TC, TG, LDL-C, ApoA1, ApoB and lower level of HDL-C were observed in pati’1,234,567/*-ents with CVD or T2DM as compared to controls.

**Table 1:** Clinical and biochemical data of the study population

Variables	CVD-		CVD+	
	T2DM- (*n* = 211)	T2DM+ (*n* = 247)	T2DM- (*n* = 232)	T2DM+ (*n* = 234)
Age (Years)	60.65 ± 11.72	61.52 ± 11.61	63.05 ± 11.79^##^	64.61 ± 10.95^&&^
Sex (male/female)	109/102	127/119	126/106^##^	119/115 ^&&^
SBP (mmHg)	124.49 ± 14.90	141.26 ± 24.43***	129.43 ± 22.18^##^	140.30 ± 24.86^&&&^
DBP (mmHg)	77.03 ± 10.75	81.60 ± 15.34***	79.32 ± 16.67	83.12 ± 14.73^&&&^
Hypertension (%)	–	96(38.8%)	59(25.4%)	145(61.9%)
Dyslipidemia (%)	–	96(38.8%)	41(17.6%)	54(23.0%)
Smokers (%)	41(19.4%)	46(18.6%)	74(31.8%)^##^	56(23.9%)
Glucose (mg/dL)	5.29 ± 1.27	14.29 ± 6.16***	5.26 ± 1.16	8.86 ± 3.34^&&&^
Triglyceride (mg/dL)	1.59 ± 0.93	2.75 ± 3.64***	1.50 ± 0.96	2.28 ± 1.75^&&&^
TC (mg/dL)	4.21 ± 1.29	5.67 ± 1.84***	5.02 ± 1.21^###^	5.00 ± 1.36^&&&^
LDL-C (mg/dL)	2.31 ± 0.88	3.12 ± 1.06***	2.81 ± 0.91^###^	2.76 ± 0.99^&&&^
HDL-C (mg/dL)	1.33 ± 0.43	1.27 ± 0.37	1.32 ± 0.34	1.17 ± 0.32^&&&^
ApoA1 (mg/dL)	1.08 ± 0.36	1.17 ± 0.33**	1.13 ± 0.28	1.09 ± 0.31
ApoB (mg/dL)	0.72 ± 0.27	0.96 ± 0.33***	0.84 ± 0.26^###^	0.88 ± 0.31^&&^

Data are presented as mean ± SD, or numbers (N) and percentage

*p-value: comparison between CVD-T2DM+ and control

^#^ *p*-value: comparison between CVD + T2DM - and control

^&^*p*-value: comparison between CVD + T2DM + and control

*SBP* systolic blood pressure, *DBP* diastolic blood pressure, *TC* total cholesterol, *LDL-C* low density lipoprotein cholesterol, *TG* triglyceride, *HDL-C* high density lipoprotein cholesterol

### APOE genotype and allele frequencies of subjects

Genotype distributions of all groups were Hardy-Weinberg equilibrium (*p* > 0.05) except the CVD + T2DM- group (*p* = 0.03). A lower ratio of E2/E2 genotype in the CVD + T2DM- group may result in deviated from the basic norms. However, this deviation may be due to contingency (Table 2). The most frequent genotype was E3/E3 in our study population. Compared with controls, the frequencies of E3/E3 and ε3 significantly decreased (*p* < 0.01 and *p* < 0.01, respectively), while E3/E4 and ε4 significantly increased in CVD patients with or without T2DM (*p* < 0.05 and *p* < 0.01, respectively). Otherwise, no significant difference was observed between T2DM + and T2DM - in the subgroup of CVD- and CVD+ (Table 3).

**Table 2:** Genotype distribution of APOE gene in different groups

Genotype	CVD-		CVD+	
	T2DM- (n = 211)	T2DM+ (n = 247)	T2DM- (*n* = 232)	T2DM+ (*n* = 234)
E2/E2	1 (0.4%)	2 (1.2%)	1 (0.31%)	1 (0.21%)
E3/E3	160 (75.8%)	183 (74.1%)	144 (41.5%)	139 (50.4%)
E4/E4	1 (0.4%)	–	6 (4.6%)	4 (1.7%)
E2/E3	24 (11.4%)	16 (6.6%)	23 (4.7%)	20 (8.3%)
E2/E4	–	3 (1.2%)	12 (3.7%)	7 (2.9%)
E3/E4	25 (11.8%)	43 (17.3%)	46 (19.8%)	63 (26.9%)
*p*-value	0.75	0.12	0.03	0.67

**Table 3:** APOE polymorphism in T2DM patients with or without CVD

Genotype	All case	CVD-		*p*	CVD+		*p*
		T2DM- (*n* = 211)	T2DM+ (*n* = 247)		T2DM- (*n* = 232)	T2DM+ (*n* = 234)	
E2/E2	5 (0.5%)	1 (0.5%)	2 (0.8%)	1.00	1 (0.4%)	1 (0.4%)	1.00
E3/E3	626 (67.7%)	160 (75.8%)	183 (74.1%)	0.67	144 (62.1.5%)^##^	139 (59.4%)^&&^	0.56
E4/E4	11 (11.9%)	1 (0.5%)	–	–	6 (2.6%)	4 (1.7%)	0.51
E2/E3	83 (8.9%)	24 (11.4%)	16 (6.5%)	0.06	23 (9.9%)	20 (8.5%)	0.63
E2/E4	22 (2.3%)	–	3 (1.2%)	–	12 (5.2%)	7 (3.0%)	0.23
E3/E4	177(19.2%)	25 (11.8%)	43 (17.4%)	0.10	46 (19.8%)^#^	63 (26.9%)^&&^	0.07
ε2	115 (6.2%)	26 (6.2%)	23 (4.7%)	0.31	37 (7.9%)	29 (6.1%)	0.29
ε3	1512 (81.8%)	369 (87.4%)	425 (86.0%)	0.53	357 (76.9%)^##^	361 (77.1%)^&&^	0.94
ε4	221 (12.0%)	27 (6.4%)	46 (9.3%)	0.11	70 (15.1%)^##^	78 (16.7%)^&&^	0.51

^#^*p*-value: comparison between CVD + T2DM- and controls

^&^*p*-value: comparison between CVD + T2DM- and controls

### Relationship between APOE polymorphism and disease

Correlation between APOE polymorphism and T2DM or CVD was evaluated using univariate analysis. Our results showed that subjects carrying E3/E4 and allele ε4 had increased risk of CVD, with unadjusted OR 1.84 (95% CI: 1.09–3.12, *p* = 0.02) and 2.71(95% CI: 1.69–4.34, *p* < 0.01), respectively. Furthermore, E3/E4 and allele ε4 appeared to increase the risk of developing T2DM patients with CVD, with unadjusted OR 2.74 (95%CI: 1.65–4.55, *p* < 0.01) and 3.05 (95% CI: 1.91–4.85, *p* < 0.01) (Table 4).

**Table 4:** Association of APOE polymorphism with the risk of T2DM, CVD, T2DM + CVD+

Genotype	Control (n = 211)	T2DM (n = 247)	Unadjusted OR (95% CI)	CVD (*n* = 232)	Unadjusted OR (95% CI)	T2DM + CVD+ (*n* = 234)	Unadjusted OR (95% CI)
E3/E3	160	183	0.91 (0.60–1.39)	144	0.51 (0.34–0.77)**	139	0.47 (0.31–0.70)**
E2/E3	24	16	0.54 (0.28–1.05)	23	0.86 (0.47–1.57)	20	0.73 (0.39–1.36)
E3/E4	25	43	1.57 (0.92–2.66)	46	1.84(1.09–3.12)*	63	2.74(1.65–4.55)**
ε2	26	20	0.64 (0.35–1.17)	37	0.86 (0.47–1.56)	29	1.02 (0.58–1.74)
ε3	369	425	0.89 (0.60–1.30)	357	0.48 (0.33–0.69)**	361	0.49 (0.34–0.70)**
ε4	26	46	1.56 (0.95–2.57)	70	2.71 (1.69–4.34)**	78	3.05 (1.91–4.85)**

*p-value: all the comparisons were made between patients and controls

Multivariate logistic regression was applied for analysis adjusting the traditional risk factors: age, sex, smoking status, SBP and DBP. It was indicated that ε4 allele was an independent risk factor for development of CVD + T2DM+ with an adjusted OR 3.69. Age and smoking was significantly correlated with increased risk of CVD and T2DM (Table 5).

**Table 5:** APOE polymorphism and the risk of T2DM and CVD represented as adjusted OR

Variables	T2DM		CVD		T2DM + CVD	
	Adjusted OR (95% CI)	*p*	Adjusted OR (95% CI)	*p*	Adjusted OR (95% CI)	*p*
ε4	1.62 (0.87–3.03)	0.13	1.78 (0.87–3.67)	0.12	3.69 (1.94–6.99)	0.00
Age	1.08 (1.05–1.11)	0.00	1.10 (1.06–1.14)	0.00	1.16 (1.12–1.20)	0.00
Sex	1.11 (0.68–1.83)	0.67	2.25 (1.11–4.56)	0.02	1.18 (0.67–2.09)	0.57
Smoking	3.39 (1.65–7.00)	0.00	7.10 (2.74–18.40)	0.00	9.64 (4.26–21.83)	0.00
SBP (mmHg)	1.03 (1.02–1.05)	0.00	1.00 (0.98–1.02)	0.84	1.02 (1.00–1.04)	0.02
DBP (mmHg)	0.99 (0.97–1.02)	0.62	1.00 (0.97–1.03)	0.83	1.01 (0.98–1.04)	0.48

After adjusting for age, sex, smoking state, SBP (mmHg), DBP (mmHg). Normal reference ranges of clinical and biochemical data are described in materials and method

### Relationship between lipid profiles and ε4 allele

We analyzed blood lipid levels in subjects with different alleles (Table 6). In the control and T2DM group, the levels of lipid showed no significant difference between E3/E3 and ε4 subjects; In the CVD group, E3/E3 patients had higher levels of TC, TG, LDL-C and ApoB than ε4 patients; In CVD patients with T2DM, we found that patients with ε4 allele have higher levels of TG and ApoA1, and lower levels of HDL-C.

**Table 6:** Comparison of fasting lipid profile between subjects carrying E3/E3 and ε 4 allele

Variables (mg/dL)	Control		T2DM		CVD		CVD + T2DM+	
	E3/E3	ε4	E3/E3	ε4	E3/E3	ε4	E3/E3	ε4
TC	4.30 ± 1.36	4.35 ± 1.19	5.58 ± 1.56	5.73 ± 2.03	4.98 ± 1.17	5.38 ± 1.21*	4.97 ± 1.36	5.20 ± 1.30
TG	1.53 ± 1.60	1.73 ± 1.65	2.51 ± 3.40	3.23 ± 3.81	1.40 ± 0.82	1.98 ± 1.33**	2.12 ± 1.49	2.81 ± 2.17**
LDL-C	2.31 ± 0.91	2.39 ± 0.91	3.15 ± 1.00	3.12 ± 1.35	2.81 ± 0.90	3.10 ± 0.84*	2.71 ± 1.00	2.99 ± 0.97
HDL-C	1.25 ± 0.43	1.24 ± 0.37	1.27 ± 0.36	1.19 ± 0.29	1.32 ± 0.32	1.23 ± 0.35	1.21 ± 0.29	1.09 ± 0.35**
ApoA1	1.03 ± 0.33	1.09 ± 0.28	1.18 ± 0.34	1.13 ± 0.28	1.13 ± 0.27	1.05 ± 0.28	1.14 ± 0.32	1.02 ± 0.29*
ApoB	0.72 ± 0.26	0.77 ± 0.33	0.97 ± 0.31	0.99 ± 039	0.83 ± 0.27	0.88 ± 0.22*	0.88 ± 0.30	0.93 ± 0.32

ε4 = E3/E4, E4/E4

Independent sample *t*-test test was applied to compare between E3/E3 and ε4 allele. Data are presented as mean ± SD

## Discussion

Apolipoprotein is a plasma glycoprotein consisting of 299 amino acid [15]. The APOE is an important gene that are associated with cardiovascular disease and diabetes. In the present study, we investigated the relationship between APOE polymorphisms and T2DM with or without CVD, and their effects on blood lipid levels.

Previous studies found that the APOE ε4 allele predicted the risk of cardiovascular events in different populations. The ε4 allele regulates the risk of coronary heart disease (CAD) in the Finnish population [16, 17]. In addition, the prevalence of CAD in diabetes patients varied according to different APOE genotypes, which was 81% in ε4 patients, 58% in E3/3 patients and 53% in E2/2 or E2/3 patients [16]. Meanwhile, the genotypes of APOE E4/4 and E4/3 were inclined to increase the risk of macrovascular disease in non-insulin-dependent DM patients [16]. The incidence of macrovascular disease, as well as lipid levels such as TC and LDL-C, was lower in patients with E2/E2 than those with other genotypes [16]. To our knowledge, ε4 allele has not been reported to affect the risk of CVD.

We are for the first time reporting the APOE polymorphism in T2DM patients with or without CVD in Hakka population. Our results indicated that E3/E3 was the most common genotype; the ε4 allele may serve as a main predictor of T2DM and CVD. A strong correlation between E3/E4 genotype and CVD in patients with T2DM was observed in our study, which was consistent with previous studies [18, 19]. After adjusting for age, sex, smoking, logistic regression analysis showed that ε4 allele increased the risk of T2DM by 1.64 times and risk of CVD by 1.80 times. Furthermore, T2DM patients carrying ε4 allele were 3.75-fold higher risk of CVD as compared to the controls (*p* < 0.01). Besides, our data suggested that age and smoking were independent risk factors of T2DM and CVD (*p* < 0.01 and *p* < 0.01, respectively).

In this study, the comparison of lipid levels between ε4 allele and E3/E3 genotype in the T2DM, CVD and T2DM with CVD patients were analyzed. It was observed that TC, TG, ApoB and LDL levels inε4 CVD patients were higher than those in E3/E3 CVD patients (*p* < 0.05). HDL and ApoA1 in ε4 T2DM with CVD patients were lower than those in E3/E3 T2DM with CVD patients (*p* < 0.05). The relationship between ε4 allele and lipid profile remains controversial [20]. ε4 allele has been shown to be associated with high levels of serum TC and LDL-C in Chinese population [21]. Dalia et al. found that subjects with E3/E4 genotypes had higher TC and non-HDL-C levels, and LDL-C levels was significantly elevated in both T2DM and CVD patients [22]. However, other lipid profiles, such as HDL-C were not correlated with APOE polymorphisms in Tunisian population [23]. In the Spanish population, women with T2DM carrying ε4 allele have higher LDL-C and lower HDL-C levels [24], suggesting that gender influences APOE polymorphism.

## Conclusion

This study preliminarily supports the fact that APOE polymorphisms are associated with T2DM and CVD. APOE ε4 allele is indicated as an independent risk factor for both T2DM and CVD. APOE genotypes are correlated with plasma lipid profiles. Other useful clinical indicators for these patients are required in Hakka population in south China.

## Data Availability

The datasets generated during the current study are not publicly available yet, due to privacy concerns and ongoing additional research. Data can be made available for peer review on reasonable request through contacting the corresponding author.

## Abbreviations

APOE: Apolipoprotein E
CAD: Coronary artery disease
CVD: Cardiovascular disease
DBP: Diastolic blood pressure
DM: Diabetes mellitus
HDL: High density lipoprotein
MI: Myocardial infarction
OR: Odds ratio
PCR: Polymerase chain reaction
SBP: Systolic blood pressure
SD: Standard deviation
T2DM: Type 2 diabetes
ε2: Epsilon 2
ε3: Epsilon 3
ε4: Epsilon 4
